# Aplastica Anemia And Viral Hepatitis

**DOI:** 10.4084/MJHID.2009.026

**Published:** 2009-12-26

**Authors:** Laura Cudillo

**Affiliations:** Stem cell Transplant Unit, Fondazione Policlinico Tor Vergata, Università Tor Vergata, Roma, Italy

## Abstract

Acquired aplastic anemia (aAA) is a severe and rare disease, characterized by hematopoietic bone marrow failure and peripheral cytopenia. The pathophysiology is immune mediated in most cases, activated T1 lymphocytes have been identified as effector cells. The disease can be successfully treated with combined immunosuppressive therapy or allogeneic hematopoietic stem cell transplantation. Hepatitis-associated aplastic anemia (HAA) is a syndrome of bone marrow failure following the development of acute seronegative hepatitis. HAA syndrome most often affects young males who presented severe pancytopenia two to three months after an episode of acute hepatitis. The clinical course of hepatitis is more frequently benign but a fulminant severe course is also described. The bone marrow failure can be explosive and severe and it is usually fatal if untreated, no correlations have been observed between severity of hepatitis and AA. In none of the studies a specific virus could be identified and most cases are seronegative for known hepatitis viruses. The clinical characteristics and response to immunotherapy indicate a central role for immune-mediated mechanism in the pathogenesis of HAA. The initial target organ of the immune response is the liver as suggested by the time interval between hepatitis and the onset of bone marrow failure. Liver histology is characterized by T cell infiltrating the parenchyma as reported in acute hepatitis. Recently in HAA it has been demonstrated intrahepatic and blood lymphocytes with T cell repertoire similar to that of confirmed viral acute hepatitis. The expanded T cell clones return to a normal distribution after response to immunosuppressive treatment, suggesting the antigen or T cell clearance. Therapeutic options are the same as acquired aplastic anemia.

## Thalassemia:

Aplastic anemia (AA) the most common bone marrow failure syndrome, is defined as peripheral blood cytopenia and a hypocellular bone marrow.

AA may develop as primary hematological disorder, most often idiopatic, or result from some causal factors as chemical toxins, drugs, viruses.

The diagnosis of AA needs an accurate differential diagnosis to exclude other causes of pancytopenia such as primary marrow diseases (myelodisplastic syndromes, acute leukaemia, lymphomas of the marrow) or non hematologic diseases (LES, sarcoidosis, deficit of folate or B12, alcohol abuse, hypotiroidism).

Bone marrow functions are usually depressed uniformly resulting in anemia, leucopenia and thrombocytopenia in peripheral blood, but less severe type of marrow failure are characterized by monocytopenia or bicytopenias or by moderate degrees of cytopenia.

The disease is infrequent, the epidemiology and incidence rate of acquired AA reported in two large controlled studies are 2/million in the West and about 2–3 fold higher in Asia^1^,^2^. This difference in geographic incidence does not have a sactisfatory explanation, probably results mainly from environmental causes rather than genetic factors^3^. The classification of AA relies on pathophysiologic pathways that identify two main forms: acquired and congenital AA. The majority of the acquired forms are idiopathic and no causative factors might be identified (Table 1).

## Pathophysiology:

Acquired Aplastic Anemia (aAA) has been associated with several factors that are responsible of qualitative and quantitative defects of hematopoietic stem cells, abnormalities of marrow stroma or defects of cell maturation and differentiation. The use of chemicals and drugs, some viral infections and autoimmune diseases have been identified as causal factors in etiology of aAA. The relationship with benzene, petrochemicals and insecticides is rarely described but well known^4^,^5^. Drugs are implicated in about 25% of aAA and can be divided into two major classes: cytotoxic drugs used in cancer chemotherapy with a known dose-dependent effect and medical drugs used in community with an unexpected idiosyncratic effect^6^,^7^. Overrepresentation of deletions in the drug-metabolizing glutathione-S-transferase genes might increase toxic drug intermediate products as observed in some series^8^. No defined mechanism has been identified for chloramphenicol, the most famous between charged agents^9^. The mechanisms that induce development of aAA after drug exposure include direct toxic effect and immune mediate destruction. The low probability of developing aAA after a drug therapy might arise from the infrequent association with enzymatic defective pathways, genetic variations of methabolism and host susceptibility to the action of toxic compound. Drug-induced aplasia does not have specific clinical features thus cannot be distinguished from idiopatic form of the disease: the clinical course, the favourable response to immunosuppressive therapy and the survival are the same as in idiopathic aAA.

Viral infections are associated with some forms of hematopoietic suppression, typically neutropenia or less frequently thrombocytopenia. Damage of hematopoietic bone marrow may be induced by direct lysis of stem cells or by immune-mediated destruction. Infectious mononucleosis due to Epstein-Barr virus (EBV) is commonly associated with some degrees of myelosuppression, more frequently neutropenia or thrombocytopenia, whereas it is only rarely complicated by AA. Pancytopenia can occur either during the acute phase or shortly after symptoms disappearance. Patients recovered spontaneously or after immunosuppressive therapy, hematopoietic recovery after antiviral treatments are also observed^10^. Parvovirus B19 infects pro-erythroblasts and leads to erythroid aplasia, but may also be associated with bone marrow failure 11. HIV infection is frequently associated with variable degrees of cytopenia.

In the last decades an unified immune-mediated mechanism with active destruction of hematopoietic stem cells by T lymphocytes has been proposed in most forms of aAA, the clinical and pathophysiologic observations have supported this hypothesis. The aberrant immune response may be triggered by drugs, virus, chemical exposure or, as in the majority of the cases, without evidence of etiologic factor^12^.

aAA shares some features with other autoimmune disorders that are characterized by T-cell mediated tissue-specific distruction. The cascade of events: cytotoxic lymphocyte activation, cytokine production and specific target organ elimination are common to all autoimmune disorders. Further the successful response of the majority of AA to immunosuppressive treatment remains the best evidence of immune pathophysiology.

The effector cells were identified by immunophenotyping as activated T cells, expressing Th1 cytokines, expecially gamma-interferon (INF-γ) and tumor necrosis factor alpha (TNF-α). Type 1 cytokines induce apoptosis in CD34 target cells, throught the FAS-dependent pathways of cell death^13^, ^14^, ^15^.

The reasons why T cells are activated in aplastic anemia is still unclear, several possible mechanisms are involved.

HLA-DR2 is overrepresented in some patients, suggesting a role for antigen recognition, it is associated with a better response to cyclosporine^16^. Polymorphisms in cytokine genes of tumor necrosis factor-α, interferon gamma and interleukin 6, related with an increased immune response, have also been observed^17^,^18^.

Another evidence of immune-mediated pathogenesis comes from a study showing that regulatory T cells (Tregs) are decreased in acquired aplastic anemia as well as in other auto immune diseases. Tregs control the development and progression of autoimmunity by suppressing autoreactive T cells^19^. A Tregs defect could explain the increased activated autoreactive T cells.

T-bet is a member of T-box family trascription factors and is responsible for the shift of CD4 T cells into the Th1 phenotype. T-bet is up-regulated in T lymphocytes from aAA patients and bounds directely to the proximal site of the INF-γ promoter, without any prior stimulation, inducing an active trascription of INF-γ gene^20^. Mutations in PRF1, the perforin gene, and mutations in SAP, encoding for small modulator protein that inhibits INF-γ production, are described in inherited and acquired aplastic anemia^21^.

The immune attack induces marrow failure secondary to reduced number and function of hematopoietic stem cells.

Stem-cell assays in vitro as evaluation of LTC-IC or cobblestone forming-cells are negatively affected, showing slow or absent colony formation^22^. Microarray of CD34+ cells from bone marrow of AA patients identified a trascriptome in which genes involved in apoptosis were up-regulated. A similar trascriptional pattern was observed in normal CD34+ cells after exposure to INF-γ^23^.

A peculiar characteristic of white blood cells in AA is short telomeres^24^. Telomere shortening may be secondary to hematopoietic stem cell replication and exhaustion, but other possible reasons of shortening have been suggested. In some patients with inherited bone marrow failure syndrome a genetic basis was also indicated for telomere deficiency. Mutations in genes that repair or protect telomeres are probably implicated in some forms of AA, but have been observed in only 10% of cases, whereas telomeres are short in about half of patients with aplastic anemia^25^. Other alternative mechanisms of repair not yet identified are probably involved in some cases.

A complication of aAA is cell clonal proliferation that coexists or appears as a late sequelae and its possible evolution to hematologic diseases such as myelodisplasia or acute leukemia. At diagnosis fifty percent of patients with aAA have expanded population of paroxysmal nocturnal hemoglobinuria (PNH) cells, detected by flow cytometry, and is a predictive marker of favourable response to immunosuppressive therapy 26. In the majority of cases PHN clones are small and no clinical manifestations are present.

Clonal caryotypic evolutions are observed at constant rate after diagnosis. The overall risk of developing a clonal cytogenetyc abnormality at 10 years is between 5% and 20% the most common cytogenetic abnormalities are aberration of chromosome 7 and trisomy 8^27^.

## Clinical Features:

Symptoms occurring in aplastic anemia result from the peripheral cytopenia and, except for those due to low blood cell count, the majority of patients did not have specific symptoms.

The findings on physical examination usually reflect the severity of pancytopenia. All of the blood cells may be depressed or a single cytopenia may be more clinically evident. Skin and mucosal bleedings at various severity degrees related to thrombocytopenia are the most common clinical manifestations. Anemia symptoms may be present, but low levels of hemoglobin can be tolerated very well without any patient’s complaints because of adaptation to a gradual reduction of hemoglobin. Granulocytopenia may induce infection, but fever and infections are uncommon at diagnosis, the typical sore throat of agranulocytosis is not often observed, probably because other alarming symptoms seek medical observation.

Acquired aplastic anemia is a diagnosis of exclusion. Clinical history and physical examination should be carefully conducted to exclude other conditions associated with pancytopenia or a secondary cytopenia. Congenital marrow failure such as Fanconi anemia (FA) can be suspected in children or teenager with bone marrow failure and some characteristic physical anomalies, the most common is skin hyperpigmentation and café au lait spots. The majority of patients are small and have short stature, malformations involving face and the upper limbs are frequent. FA is a chromosome instability syndrome characterized by hypersensitivity to interstrand DNA crosslinking agents as di-epoxibutane (DEB) and mitomycin C. Chromosomal breakage test with DEB show an excessive chromosomal breakage in peripheral blood lymphocytes from FA patients.

Laboratory screening and data are also irreplaceable tools to identify other cause of cytopenia and more to grade the severity of aplastic anemia as defined by peripheral blood cell counts. (Table 2)

Examination of the bone marrow is basic for the diagnosis of AA, the marrow must be assessed quantitatively and qualitatively for cellularity and morpholology of hematopoietic cells.

Marrow aspiration smear and bone marrow biopsy specimen should be performed always, an adeguate sample is required for the diagnosis and sometime it may be necessary to repeat a second procedure. Marrow examination should include flow-cytometry for phenotypic analysis to exclude acute leukemia and myelodisplasia and evaluation of glycophosphoinositol-anchored proteins to diagnose PNH. Bone marrow cytogenetics is diagnostically important, kariotype of marrow cells is usually normal in AA, but frequently a clonal abnormality is observed in myelodisplastic syndromes.

## Treatment of Acquired Aplastic Anemia:

The therapeutic choice is based on aplastic anemia severity, thus on peripheral blood cell count. Patients with moderate cytopenia not requiring regular transfusions can receive supportive therapy with low dose steroid, or cyclosporine in combination with ATG, but few controlled studies have addressed moderate aAA^28^. Androgen may be effective in presence of residual hematopoietic function. In vitro androgens increase the telomerase activity in human lymphocytes and CD34 cells, this action probably supports the recovery of hematopoiesis^29^.

Patients with severe aplastic anemia can be offered combined immunosuppressive treatment (IST) or hematopoietic stem cell transplantation (HSCT).

Antitymocyte globulin (ATG) and cyclosporine (CSA) is the most common immunosuppressive regimen, based on the outcome of large studies^11^,^30^,^31^. Both horse and rabbit ATG have been used, ATG are produced by immunizing animals (rabbit or horse) against human thymocyte and its activity relies on immunomodulation and lymphotoxicity associated to a preferential depletion of activated T cells. The larger experience is with ATG produced in horse, although both horse and rabbit ATG have been used successfully.

Frickhofen et al.^32^, comparing treatment with ATG plus CSA versus ATG alone in long term follow up, showed a difference in event free survival, indicating a higher rate of relapse in patients given ATG alone requiring additional IT course; however at 10 years survival no difference was observed

Time of response to therapy with ATG plus CSA is 120 days as median, where response can be defined as complete (CR) and partial (PR): CR relies on normal peripheral blood count, PR requires a transfusion independence.

Global response rates (CR plus PR) are about 60% as reported from several studies^32^,^33^,^34^. In a study^30^ from the EBMT Severe Aplastic Anemia Working Party on almost 1000 patients the most important negative prognostic factors on survival was age (older than 16 years) and interval time between diagnosis and therapy over 23 days. Neutrophil count, in contrast with a previous study^35^, had no influence on survival. Relapse is defined as renewed need for transfusion in patient who achieved previous transfusion independence for at least 3 months and occurring in about 30% of patients. Relapse can be successfully treated with a second course of IST^36^,^37^.

In large randomized studies the addition of G-CSF improved neutrophil count, but not survival at 3 years^38^,^39^; furthermore two retrospective studies show an increased risk of late clonal disorders in patients receiving G-CSF^40^,^41^ Data from a recent meta-analysis confirms that hematopoietic growth factors are not recommend in aAA^42^.

Allogeneic Hematopoietic Stem Cell Transplantation (HSCT) replaces hematopoietic and immune system curing the majority of patients. Standard conditioning consisted of cyclophosphamide 200mg/m^2^ with ATG, this preparative regimen does not cause infertility or second tumours and offers an excellent response rate^30^. An IBMTR study showed 77% 5-years survival in matched sibling donor transplantation, in children and patients allograft who are minimally transfused improved results have been observed and survival ranged from 80% to 90%^43^.

A report from EBMT analyzed more than 1500 patients receiving allogeneic transplantation. Favorable prognostic factors on survival were: years of transplantation after 1997, a matched sibling donor, an age younger than 16 years, an interval between diagnosis and transplant of less than 83 days and a conditioning regimen without radiation. The survival for patients younger than 16 years is 91%, but for older patients the results are less encouraging with survival of 51% in patients aged over 40 years 30.

A matched sibling donor is available in about 30% of cases. The outcome is poor in patients who have failed IS, thus in such patients a transplant from matched unrelated donor (MUD) is a suitable treatment. Data from the European Registry show a rejection rate of 15%, an incidence of acute GVHD of 48% and survival at 5 years estimated at 39% in HSCT from MUD performed from 1988 to 1998^44^. Significant progress has been made in recent years addressing the issue of conditioning regimen, but also the degree of class I HLA match and the time interval from diagnosis are important. A retrospective analysis from the Japanese Registry suggested that in patients with most favourable characteristic and conditioned with low dose radiation survival is comparable with HSCT from matched sibling donors^45^.

Bacigalupo et al. for the EBMT Aplastic Anemia Working Party obtained encouraging results employing a non-radiation-based conditioning with 70% of survivors, although reject was high in adults older than 14 years^46^.

In conclusion the treatment of AA has improved dramatically over the last years and a long term survival of more than 75 % can be achieved with either IST or HSCT.

## Hepatitis-Associated Aplastic Anemia:

Hepatitis-associated aplastic anemia (HAA) is a variant of aplastic anemia in which aplastic anemia follows an acute seronegative hepatitis. This syndrome was first described in two patients in 1955 and from 1975 more than 200 cases have been documented^47^,^48^.

HAA is not uncommon, with an episode of hepatitis preceding the onset of bone marrow failure in 2% to 5% of cases of AA in Europe and United States^49^, the incidence is higher in Asia ranging from 4 to 10%, where it is associated with low socioeconomic status, rice farming and agricultural pesticides exposure^50^.

Development of AA is also described following orthotopic liver transplant (LTx) for viral seronegative hepatitis. In a multicentric review study of 32 patients undergoing LTx for acute non-A non-B hepatitis nine (28%) developed AA 51. In another study aplastic anemia developed in 33 % (6 of 18) of children who required liver transplantation for fulminant hepatitis^52^. This high incidence contrasts with the low incidence of AA, less than 1%, reported after LTx for other causes and strongly suggests that the etiologic agent of hepatitis is probably involved also in the pathogenesis of AA after transplant.

HAA syndrome has some typical clinical features: most often affects young males who presented severe pancytopenia two to three months after an episode of acute hepatitis. Hepatitis presents variability in clinical features but generally follows a benign course, showing a partial or complete resolution before AA onset. The bone marrow failure can be explosive and severe and it is usually fatal if untreated, no correlations have been observed between severity of hepatitis and AA.

The hepatitis was clinically indistin-guishable from a typical viral hepatitis, but in no study a specific virus was identified and most cases are seronegative for known hepatitis viruses, including A, B, C and G virus. HGV and TTV have been suspected as agents of hepatitis but no study confirms this relationship^53^,^54^. SEN virus is a circovirus with eight different variants, SENV-D and SENV-H were found in more than 50% of transfusion-associated hepatitis, but the etiologic role in liver damage is not clear. In patients with HAA SEN viruses D and H were detected by PCR amplification, but patients probably acquired the virus from transfusions, thus the relationship between SENV and HAA remains to be established^55^.

The current treatments of HAA are supportive therapy, IST and allogeneic HSCT, as in the setting of others forms of AA.

A retrospective analysis of ten cases of HAA referred by the Clinical Center at the National Institutes of Health (NIH) did not show evidence of active hepatitis in the sera of patient by serologic or PCR evaluations. All patients had evidence of severe AA and presented elevated liver enzyme levels. They were treated with immunosuppressive treatment which consisted of a four-day course of intravenous antithymocyte globulin and oral cyclosporine for 6 months, seven patients responded. Liver enzyme returned to normal range within one month after therapy, hematologic recovery was slower, occurring after at least six months. At the presentation all patients had activated CD8 lymphocytes in peripheral blood, after immunosuppressive treatment CD8 cells decreased in all patients but one who did not respond, suggesting that liver and bone marrow associated damages are immune-mediated^54^.

Osugi et al. evaluated the efficacy of ATG and CSA in 44 children with HAA. Response rate at 6 months was 70%, no hepatic toxicity related to therapy was observed and a rapid decrease in ALT levels were reported^56^.

Another study assessed the efficacy of allogeneic bone marrow transplantation in patients with HAA^53^. None had evidence of hepatitis virus A, B, C, E, and G at the time of diagnosis. Seventeen patients underwent BMT from HLA-matched donors, twelve were alive at mean follow up of 38 months; survival and transplant related mortality were similar to those patients transplanted for idiopathic AA, no cases of recurrent hepatitis occurred during the follow- up period.

A report from EBMT Severe Aplastic Anemia Working Party showed that previous hepatic damage from viral hepatitis and liver functions abnormalities did not appear to increase the toxicities from hepatocellular damage in the post transplant course^57^.

The clinical characteristics and response to immunotherapy indicate a central role for immune-mediated mechanism in the pathogenesis of HAA. The initial target organ of the immune response is the liver as suggested by the time interval between hepatitis and the onset of bone marrow failure.

In acute B and C hepatitis and in autoimmune hepatitis liver histology is characterized by T cell infiltrating the parenchyma. Analysis of T cell repertoire in these cases has demonstrated clonotypic expansions in T Cell Receptor (TCR) Vβ subfamilies, indicating a high restriction in the T cell composition of lymphocytes infiltrating the liver^58^.

Lu investigated the T cell repertoire of intrahepatic lymphocytes on HAA patients and reported that there is an infiltration of both clonal and many non clonal T cells with a T-cell repertoire similar to that of both viral B and C and autoimmune-hepatitis^59^. The expanded T cell clones return to a normal distribution after response to immunosuppressive treatment, suggesting the antigen or T cell clearance.

## Conclusion:

HAA is a life-threatening condition requiring prompt treatment. It can be considered a subset of AA as suggested by pathogenetic immune mediated mechanism and by response to immunosuppressive therapy. The therapeutic options are allogeneic HSCT and IST, outcome and response rate are similar to those for patients with idiopathic acquired aplastic anemia.

## Figures and Tables

**Table 1.** Classification of aplastic anemia.

***Acquired***	
Idiopatic	
Secondary	Irradiation Drugs and chemical: cytotoxic agents, benzene, chloramphenicol, gold salts, nonsteroidal anti-inflammatory drugs Idiosyncratic reactions Viruses: Epstein-Barr virus, hepatitis virus unidentified, Parvovirus B19, HIV Immune diseases Pregnancy Paroxysmal nocturnal hemoglobinuria
***Inherited aplastic anemia***	Fanconi’s anemia Dyskeratosis congenital Amegakaryocytic thrombocytopenia Shwachman-Diamond syndrome

**Table 2.** Grading of Aplastic Anemia

**Severe aplastic anemia**	

Bone marrow cellularity	<30%

Two of three peripheral blood criteria:	
-absolute neutrophil count	500- 200/mm3
-platelet count	<20.000/mm3
-reticulocyte count	<40.000/mm3

**VERY SEVERE APLASTIC ANEMIA**	
-absolute neutrophil count	<200/mm3

**MODERATE APLASTIC ANEMIA**	
Patients with pancytopenia who do not fitfull the criteria of severe disease	
